# Clear aligner treatment: different perspectives between orthodontists and general dentists

**DOI:** 10.1186/s40510-019-0263-3

**Published:** 2019-03-11

**Authors:** Fabrizia d’Apuzzo, Letizia Perillo, Caroline K. Carrico, Tommaso Castroflorio, Vincenzo Grassia, Steven J. Lindauer, Bhavna Shroff

**Affiliations:** 1 1 Orthodontic Division, Multidisciplinary Department of Medical-Surgical and Dental Specialties, University of Campania “Luigi Vanvitelli”, Via Luigi De Crecchio 6, 80138 Naples, Italy; 2 Department of Periodontics, School of Dentistry, Virginia Commonwealth University, Richmond, VA USA; 3 Department of Orthodontics, Dental School, University of Turin, Turin, Italy; 4 Department of Orthodontics, School of Dentistry, Virginia Commonwealth University, Richmond, VA USA

**Keywords:** Aligners, Orthodontists, General dentists, Malocclusion, Patients’ perception

## Abstract

**Purpose:**

To evaluate differences between orthodontists and general dentists in experience with clear aligners (CA), patients’ demand and perception, types of patients, and malocclusion treated with CA and to compare the two groups of clinicians not using CA in their practice.

**Methods:**

A Web-based survey was developed and sent to the 129 members of the European Aligner Society and randomly to 200 doctors of dental surgery by e-mail. They responded on demographics and to one of two different parts for clinicians using CA or not using CA. Statistical analysis was performed with SAS EGv.6.1.

**Results:**

The response rate was 74%. Among the total of respondents, the majority reported utilizing CA in their practice with a greater percentage of orthodontists (*P* = 0.0040). Overall, orthodontists learned more about CA during academic seminars comparing to general dentists, and they treated more class I with crowding (*P* = 0.0002) and with open bite (*P* = 0.0462). The majority of patients treated with CA were female and adults with a full-time employment, and the patients’ knowledge about CA treatment was mainly provided by information from external media advertising. For respondents not using CA, orthodontists were more likely to report that CA limit treatment outcomes, whereas general practitioners were reported not having enough experience to use them.

**Conclusions:**

There were some significant differences between orthodontists and general dentists mainly in experience and case selection for clinicians using CA as well as in the reasons provided for not using CA in their practice.

**Electronic supplementary material:**

The online version of this article (10.1186/s40510-019-0263-3) contains supplementary material, which is available to authorized users.

## Background

Clear aligners (CA) have been used in orthodontics since 1946 when Dr. Harold Kesling introduced the use of a series of thermoplastic tooth positioners to obtain tooth alignment [1]. CA treatment has evolved mainly over the last 15 years through new technologies and materials to widen the range of tooth movements [2]. The main advantages of CA treatment are better esthetics with higher patient acceptance and a general better quality of life [3]. CA treatment causes less pain compared to a traditional fixed treatment [4] and also an improvement of the gingival and periodontal health indexes. The treatment with CA is usually performed in combination with other orthodontic auxiliaries and procedures such as attachments, interarch elastics, and interproximal reduction [5]. However, there are some significant limitations in treating complex malocclusions, i.e., the limited root-movement control, the intermaxillary discrepancy correction, the anterior extrusion, and rotation movement [6–8]. Moreover, the reliance on patient compliance has been also reported as an important variable for the CA treatment outcome [9, 10]. The clinicians who want to use CA to treat their patients have to rely on their own clinical experience, expert opinions, and limited published evidence-based results [11–14]. CA can be provided by both orthodontists and general dentists; however, some significant differences were evinced between the two groups in the use of a CA treatment in their clinical practice [15]. Several differences in treatment plan and management, as well as training and expertise between orthodontists and general dentists performing Invisalign® treatments, were also found in a recent survey [16]. To date, no information on the differences between orthodontists and general dentists’ experience with CA, case selection, type of clinical practice, and patients were provided. Moreover, an assessment of clinicians not providing CA treatment in their clinical practice and their future perspective was never performed. Therefore, the purposes of this survey were to evaluate the differences between orthodontists and general dentists in their experience and types of dental malocclusion treated with CA, the patients’ demographics, demand and perception of CA treatment, and to compare the two groups of clinicians actually not using CA in their practice with an evaluation of reasons provided for this choice.

## Methods

Approval to conduct this study was obtained from the Institutional Review Board of the University of Campania “Luigi Vanvitelli”, Naples, Italy (N. Prot. 1030).

A Web-based survey was developed for orthodontists and general dentists to respond to statements about the perspective of the clear aligner treatments. The online surveying software REDCap (Research Electronic Data Capture) hosted at the Virginia Commonwealth University, Richmond VA, USA, was used to collect data in this study. This electronic surveying tool was configured to collect subject survey responses anonymously. Prior to the beginning of the study, an expert panel composed of three orthodontists and three general dentists from the University of Campania “Luigi Vanvitelli” and an expert in survey research from the Virginia Commonwealth University reviewed the survey questionnaire. Feedback obtained from this group was used to modify the survey for content and validity, and the final version was redacted (see Additional file 1).

The European Society of Aligners (EAS) delivered by e-mail an invitation letter with a link of the Web survey to all the 129 members of the society. The survey was also sent out randomly by e-mail to 200 doctors of dental surgery (DDS) from the academic mailing list. A second reminder was sent out 2 weeks later to increase participation. Because there was no way to track who responded to the first e-mail, the recipients were asked in the second e-mail to not participate if they had already performed the survey. The data were collected over the course of 8 weeks, from May 5 to June 30, 2017. The front page of each survey contained the title of the study and briefly explained its purposes. At the beginning of the survey, respondents were asked whether they were orthodontists (with a specialty or a recognized degree in orthodontics) or general dentists in order to allow group comparisons of the results. A common section of the survey gathered demographic information, such as gender, age, years in practice, and type of work. Respondents were then asked if they use the clear aligners orthodontic treatment in their clinical practice. After this initial series of statements, the invited participants were triaged to respond at one of two different parts of the survey: the first for clinicians using CA and the second for clinicians not using CA. The survey section for CA users contained statements regarding the personal experience with CA treatment, such as the years using CA, the number of cases started in the last 12 months, and the individual learning on the use of CA. Then, providers responded questions on the patients more often requiring an orthodontic treatment with CA. In particular, they were asked data about patients’ gender, age, occupation status, and perception on the CA treatment. In the last section of the questionnaire for users of CA, they were asked information about the main type of patient (teen, adults, periodontal patients, pre-prosthetic or craniofacial patients) and about the dental malocclusion they were more willing to treat with CA.

The survey section for clinicians not providing CA treatments in their clinical practice collected information about the main reasons for not using CA in the two groups of dental practitioners and their perspectives for a potential use in the next future.

### Statistical methods

Responses were summarized using descriptive statistics. Not all respondents answered all questions; all available responses were analyzed, and the unanswered questions were excluded from further analysis. Chi-squared tests were used to test for differences between the two groups of respondents, orthodontists, and general dentists. SAS EG v.6.1 was used for all analyses. The significance level was set at 0.05.

## Results

A total of 245 respondents on a total of 329 completed the questionnaire, 188 orthodontists (77%), and 57 general practitioners (23%). The response rate was 74%.

In terms of demographics, respondents represented 25 countries, the majority was from Italy (69%) and other countries included UK (4%), Switzerland (3%), France (3%), and Greece (3%).

There was an equal distribution of gender between orthodontists and general dentists (males 51% vs. females 49%, *P* > 0.9). The age of the respondents ranged between 24 and more than 61 years; 14% of orthodontists versus 31% of general dentists ranged between 24 and 30 years, and 50% of general dentists were in practice since 1 to 10 years, thus general dentists were more likely to be younger than orthodontists (Table 1). The majority worked at a private single practice (57%), and this was similar between the two groups (*P* > 0.8) (Table 1).

**Table 1:** Differences between orthodontists and general dentists in demographics

Demographics	Orthodontists (%)	General dentists (%)	*P* value*
Gender (*n* = 217)			0.9776
Male	51	51	
Female	49	49	
Age (*n* = 219)			*0.0375*
24–30	14	31	
31–40	32	20	
41–50	25	27	
51–60	23	14	
61+	6	8	
Years in practice (*n* = 216)			0.2045
1–10 years	35	50	
11–20 years	26	25	
21–30 years	27	19	
31–40 years	12	4	
41+ years	0	2	
Practice type (*n* = 189)			0.8851
University/hospital academic staff	7	6	
Private practice solo	56	60	
Private practice team/multidisciplinary	36	34	

*P value from chi-squared tests

Among the total of respondents, 79% reported currently utilizing clear aligners in their practice, with a greater percentage of orthodontists than general dentists (83% vs. 65%, *P* = 0.0040). However, among the clear aligner users, the number of cases treated was similar between general dentists and orthodontists. In particular, the higher percentage of respondents was using CA for the past 5 years with no more than 10 cases started in the last 5 years (Table 2).

**Table 2:** Differences between orthodontists and general dentists in experience with CA treatment

	Orthodontists (%)	General dentists (%)	*P* value*
CA users (*n* = 191)	83	65	*0.0040*
Years using CA (*n* = 190)			0.2086
1–5 years	55	67	
6–10 years	24	25	
11–15 years	21	8	
No. of CA cases started in the last 12 months	(*n* = 199)		0.7423
1–10 cases	45	57	
11–20 cases	19	4	
21–30 cases	8	9	
31–40 cases	4	0	
41–50 cases	4	3	
51+ cases	19	14	

*P value from chi-squared tests

Providers gained information about clear aligners through private courses (68%), academic seminars (49%), congress lectures (42%), and book chapters or papers (35%). Orthodontists learned more about CA during academic seminars (53% vs. 35% of general dentists, *P* = 0.00564), whereas general dentists attended more private courses on CA (78%) (Table 3).

**Table 3:** Comparison between orthodontists and general dentists in learning about the use of CA

Type of learning about CA (*n* = 191)	Orthodontist (*n =* 154) (%)	General dentist (*n =* 37) (%)	*P* value*
Academic seminars	53	35	0.0564
Private courses	65	78	0.1168
Congress lectures	44	35	0.3541
Books and/or papers	37	27	0.2531
Other	6%	5%	0.8065

*P value from chi-squared tests

**Fig. 1:**
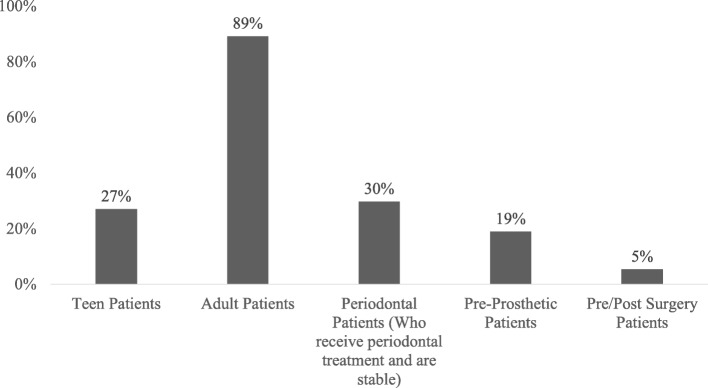
Type of treated patients

A majority of adults was treated with CA (97%) and a good percentage of patients treated with CA (30%) presented with previous periodontal disease in a stable phase to proceed with orthodontic therapy (Fig. 1).

The type of cases most providers, both orthodontists and general dentists, reported in treatment with clear aligners were class I dental relationships with spacing (75%) or crowding (86%) (Table 4).

**Table 4:** Description of clear aligner cases treated by the total number of respondents

	Number	Percentage
Treated cases		
Class I spacing	143	75
Class I crowding	165	86
Class I deep bite	118	62
Class I open bite	90	47
Class II deep bite	105	55
Class II open bite	63	33
Class III deep bite	46	24
Class III open bite	38	20

In comparing between orthodontists and general dentists, there were no differences in case selection except for class I with crowding (91% orthodontists vs. 68% general dentists, *P* = 0.0002) and class I with open bite (51% orthodontists vs. 32% general dentists, *P* = 0.0462) that were treated by a higher number of orthodontists (Table 5). About the half of the respondents, both orthodontists and general dentists were willing to treat a malocclusion with a moderate crowding (4–6 mm), and no differences were found between the two provider groups (Table 5).

**Table 5:** Comparison between orthodontists and general dentists in malocclusion treatment

	Orthodontists (%)	General dentists (%)	*P* value*
Treated cases			
(*n* = 143) Class I spacing	76	70	0.4726
(*n* = 165) Class I crowding	91	68	*0.0002*
(*n* = 118) Class I deep bite	64	51	0.1460
(*n* = 90) Class I open bite	51	32	*0.0462*
(*n* = 105) Class II deep bite	57	46	0.2190
(*n* = 63) Class II open bite	34	27	0.3907
(*n* = 46) Class III deep bite	25	19	0.4132
(*n* = 38) Class III open bite	22	11	0.1232
Crowding (mm) (*n* = 185)			0.9878
(*n* = 48) 1–3 mm	25	28	
(*n* = 91) 4–6 mm	50	47	
(*n* = 24) 7–9 mm	13	13	
(*n* = 22) > 10 mm	12	13	

*P value from chi-squared tests

Patients requiring a treatment with clear aligners were mainly females (74%) with an age between 18 and 45 years. Sixty-nine percent of them were employed full-time, and a majority (73%) was already informed about the CA treatment and requested for this type of treatment (Table 6).

**Table 6:** Patients’ characteristics and perception of the total number of respondents

	Number	Percentage
Gender distribution of patients		
Equal males and females	75	40
More males than females	10	5
More females than males	101	54
Age of patients requesting CA		
< 18	21	11
18–30	134	70
31–45	139	73
46–60	38	20
> 60	13	7
Occupational status of patients		
Students	12	6
Full-time employed	128	69
Part-time employed	12	6
Unemployed	2	1
Retiree	3	2
I do not know	29	16
Patients’ reasons for CA treatment request		
Information by advertising and directly asking for CA	139	73
Information through the office marketing	59	31
Word of mouth from friend or family member	77	40
Suggestion of the doctor	96	50

**Fig. 2:**
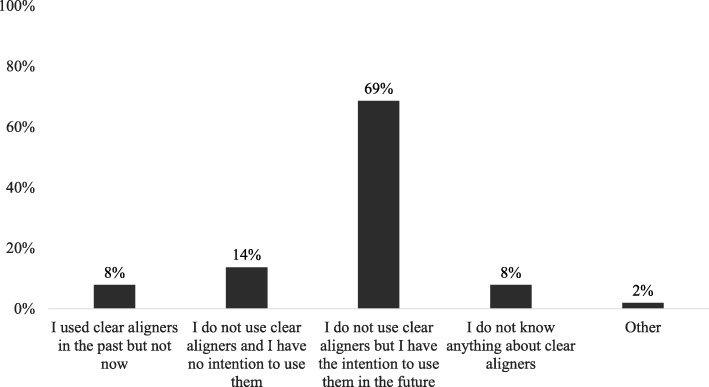
Summary of respondents who reported not utilizing CA

**Fig. 3:**
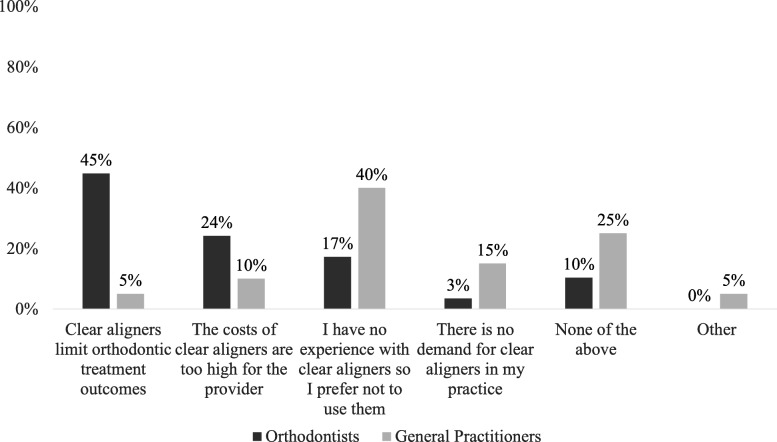
Comparison between orthodontists and general dentists in reason for not utilizing CA

The majority of respondents who reported not using clear aligners declared the intention to begin using them in the next future (69%) (Fig. 2). There were significant differences in the reasons for not utilizing clear aligners based on the practitioner type (*P* = 0.0099). Orthodontists were more likely to report that clear aligners limit treatment outcomes (45% vs. 5%), whereas general practitioners were reported not having experience with clear aligners (40% vs. 17%) (Fig. 3).

## Discussion

The clear aligner treatment in the last years has been increasing its relevance and use. The published papers comparing CA with conventional fixed appliances mainly show flaws and lacks including poor methodology with a high risk of bias, the absence of control group or blinding procedures, and small sample size affecting the internal validity and the outcomes of the studies [17].

This questionnaire collected information on the effective use and management of CA focusing not only on differences between orthodontists and general dentists, but also on the type of patients demanding the invisible orthodontic treatment with CA.

The questionnaire was responded by a higher number of orthodontists than general dentists maybe because the topic regarding a specific field of orthodontics, the clear aligner treatment, was well specified in the title of the survey and it has aroused more interest among the specialists in orthodontics than in general dentists. Seventy-nine percent of total respondents was using CA in their practice, and the main part of them was orthodontists (*P* = 0.004) as expected considering the topic. To note, the main part of general dentists who answered the questionnaire declared to use CA in their practice (65% vs. 35% not using CA), thus only dentists having a previous knowledge on clear aligner treatment seemed to be interested in the survey and accepted to perform it.

Fifty-seven percent of CA users treated less than 10 cases in a year and 67% used CA since less than 5 years. These results maybe depend on less confidence in using a technique different from the conventional multibracket fixed appliances in which the reliability of treatment outcomes and patient compliance were better demonstrated in literature for many decades. The orthodontists responding to the survey seem to have overall more experience than general dentists with more CA cases started in the last 12 months, although the comparison between the two provider groups was not statistically significant. General dentists indicated that information about clear aligners was mainly gained through private courses and less from academic seminars, congress lectures, and book chapters or papers in comparison to the orthodontists, which are conversely more related to the academic environment after their postgraduate program (Table 3). Thus, the interest in the CA treatment seems more widespread among the younger clinicians to whom this technique is taught at postgraduate orthodontic programs or that regularly participate in seminars and congress lectures.

The adults represented the higher percentage of patients treated with CA by both orthodontists and general dentists (97%), and this trend is in agreement with previous studies [2, 4, 6]. Moreover, the treatment with CA was often used in patients with periodontal problems (Fig. 1). Rossini et al. [18] concluded that the periodontal health indexes were significantly improved during CA treatment. Other studies comparing CA with conventional fixed appliances showed that patients treated with clear aligners presented a better periodontal status evaluated by plaque index (PI), pocket probing depth (PD), and the bleeding probing (BOP) compared to patients treated with fixed appliances because of the facilitated oral hygiene with the removable appliances [19]. However, a good oral hygiene education and continuous and repeated professional tooth cleansing are mandatory to get successful combined periodontal and orthodontic treatment [20].

Other categories of subjects treated by the respondents with CA were patients for which a pre-prosthetic orthodontic treatment was required (19%). The treatment with CA may be indicated to plan and visualize the treatment outcomes before starting the multidisciplinary treatment [7]. The teens represented the 27% of patients treated with CA by the respondents. Younger patients are increasingly involved in CA treatment in the last years, although this was not the original idea of this treatment solution [1]. This is maybe due to the major request of invisible orthodontic treatment even in younger patients with a greater awareness of oral health and a consequent greater adherence during active orthodontic treatment with removable appliances. About the orthodontic treatment before and after jaw surgery, the 11% of respondents considered the use of CA. In the literature, there are a few clinical reports in which treatment with CA was combined with surgery in severe cases [21, 22]. However, the survey outcomes demonstrate the widened perspective of CA use even in complex clinical cases.

Overall, the higher percentage of both orthodontists and general dentists reported they were more confident treating class I dental relationships and malocclusions with a mild-to-moderate crowding (Table 4). This confirms previous findings that have shown good confidence of the practitioners in treating a mild crowding than a severe one [15, 16]. Significant variations between the two groups were reported in the treatment of class I with crowding or class I with open bite that orthodontists seemed to be more inclined to treat (Table 5). In comparing data with previous results of Best et al. [16], a major number of orthodontists declared to treat class I and II malocclusions with deep bite.

About the patients’ characteristics, the respondents revealed that CA treatments were mainly performed in females than males (54% females vs. 5% males) (Table 6). This was expected according to previous studies [4, 11]. Moreover, the study of Jeremiah et al. showed that the social interactions and well-being of a young female adult are influenced by a visible orthodontic appliance with no affection by gender of the judges, whereas an appliance with more positive social judgments would be deemed best for social acceptance [23].

The respondents said that the patients demanding for an invisible treatment with CA were mainly aged between 18 and 45 years, as also showed in previous studies [4, 24]. Older adults with more than 45 years represented a good percentage of patients (27%) treated with CA by the respondents, maybe because the functional and esthetical concern is increasing together with the longer and better life in the developed countries (Table 6). The majority of patients were employed full-time, and this was expected to afford the greater CA treatment fees in comparison to conventional labial fixed orthodontic appliances [24].

A high percentage of patients wearing CA seemed to have previously received information about this type of treatment by advertising on social media and network (73%). A lower percentage received suggestions from the doctor himself, or from word of mouth of friend or family member, and in a little percentage through the office marketing (Table 6). Recent studies have underlined the increased relevance of marketing and social media in our working activities. Orthodontists and patients routinely get access to social media and practice websites that are indeed becoming effective marketing and positive communication tool in the orthodontic practice and patient experience [25–27]. Twenty-one percent of respondents declared to not use clear aligners in their practice and answered a different series of questions, and the main part of them (69%) would begin using them in the next future (Fig. 2). A little part of them reported to have used CA only in the past and to have no further intention to consider CA as an orthodontic treatment option in their practice. A little percentage of respondents, but still to remark (8%), declared to not know anything about clear aligners, maybe because of younger age or a few years in practice.

The major part of orthodontists reported to not use CA because of the limited orthodontic final treatment outcomes, the higher price in comparison to traditional fixed appliances or the personal less experience.

The insight into the type of malocclusions and patients demanding for CA in the clinical practice was an original point of this survey, and it was underlined which increasingly focus on the esthetic appearance of adults and the importance of advertising and marketing in the great interest in invisible orthodontic treatment with CA. This survey also focused on practitioners not using clear aligners in their practice and the different reasons between the two groups.

A limitation of this study is that the respondents to the survey were more orthodontists than general dentists. The country more represented was Italy, as could be expected considering the sample recruitment method used in the study. Future studies could be performed to widen the sample and to analyze the different treatment duration and outcomes between the two categories of practitioners. Another interesting aspect could be to predict the best patient to treat with CA according to his compliance and motivation.

## Conclusions

- Orthodontists were using clear aligners for more years and had started more cases in the previous 12 months than general dentists.
- Orthodontists learned about clear aligners mostly during academic seminars, congress lectures and papers or books in comparison to general dentists.
- The types of malocclusion mainly treated from both orthodontists and general dentists were class I spacing and class I with crowding. In comparing the two groups, a higher percentage of orthodontists treated class I with crowding and class I with open bite.
- The majority of patients (97%) treated with clear aligners were adults between 18 and 45 years, mainly females, with a full-time employment.
- The patients’ knowledge about the clear aligner treatment was mainly provided by information from external media advertising.
- The main part of respondents not currently using clear aligners in their practice was willing to use them in the future (69%).
- Forty-five percent orthodontists not using clear aligners considered the outcomes with this type of treatment limited compared to conventional fixed appliances, whereas 40% of general dentists were not using clear aligners because of their poor experience.

## Additional file

Additional file 1: Final version of the Questionnaire. (PDF 122 kb)

## Abbreviations

CA: Clear aligners
DDS: Doctors of dental surgery
