# Supplementary material for: Clear aligner treatment: different perspectives between orthodontists and general dentists
Source: Prog Orthod. 2019 Mar 11;20:10. doi: 10.1186/s40510-019-0263-3 (PMC6409290; doi:10.1186/s40510-019-0263-3)
Supplement: Supplementary file 1 — Final version of the Questionnaire. (PDF 122 kb) [file 40510_2019_263_MOESM1_ESM.pdf]

Dear Colleague,
Thank you very much for your willingness to participate in this survey on the clear aligner orthodontic treatment. Please, complete the questionnaire below.

CLEAR ALIGNERS - QUESTIONNAIRE

1. **Are you a…?**
   - Orthodontist (with a Specialty or a recognized Degree in Orthodontics)
   - General dentist

2. **Do you currently use the clear aligners orthodontic treatment in your practice?**
   - Yes
   - No

*[if you answered **Yes**, please go to question number 3 to 17;*
*if you answered **No**, please go to question number 18]*

CLEAR ALIGNER TREATMENT MANAGEMENT

3. **How many years have you used the clear aligners?**
   - 1-5 years
   - 6-10 years
   - 11-15 years

4. **How many clear aligner orthodontic cases were started in your practice during the last 12 months?**
   - 1-10 cases
   - 11-20 cases
   - 21-30 cases
   - 31-40 cases
   - 41-50 cases
   - > 50 cases

5. **How did you learn to use clear aligners? (Check all that apply)**
   - In academic seminars
   - In private course
   - In congress lectures
   - In books and/or papers

6. **What type of practice do you have? (Check all that apply)**
   - University
   - Hospital
   - Private practice solo
   - Private practice team/multidisciplinary
   - Full time (4 days or more)
   - Part time (3 days or less)

7. **In which of the following patients do you prefer to use clear aligners?**
   - Teen patients
   - Adults patients
   - Periodontal patients (who received periodontal treatment and that are stable)
   - Pre-prosthetic patients
   - Pre/post-surgery patients

8. **How much dental crowding do you usually correct with clear aligners?**
   - 1-3 mm
   - 4-6 mm
   - 7-9 mm
   - $\geq$ 10 mm

9. **What do you prefer to correct with the clear aligners? (Check all that apply)**
   - Class I spacing
   - Class I crowding
   - Class I deep bite
   - Class I open bite
   - Class II deep bite
   - Class II open bite
   - Class III deep bite
   - Class III open bite

TYPE OF PATIENTS INTERESTED AND/OR TREATED WITH CLEAR ALIGNERS

10. **What is the higher patient gender percentage that you treat with clear aligners?**
    - Male
    - Female

11. **What is the age of patients more interested in clear aligner treatment?**
    - < 18 years
    - 18-30 years
    - 31-45 years
    - 46-60 years
    - $\geq$ 60 years

12. **What is the occupation status of the patients interested in clear aligner treatment?**
    - Students
    - Full-time employed
    - Part-time employed
    - Un-employed
    - Retiree
    - I don't know

13. **How did the patient choose a clear aligner treatment? (Check all that apply)**
    - The patient was already informed by advertising and directly ask for clear aligner treatment

- The patient learn this treatment option through the office marketing products
- The doctor suggests it

DEMOGRAPHIC CHARACTERISTICS, TYPE OF PRACTICE AND BACKGROUND OF THE SURVEY PARTICIPANTS

14. **What is your gender?**
    - Male
    - Female

15. **What is your age?**
    - 24-30 years
    - 31-40 years
    - 41-50 years
    - 51-60 years
    - ≥ 61 years

16. **How many years have you been in practice?**
    - 1-10 years
    - 11-20 years
    - 21-30 years
    - 31-40 years
    - > 40 years

17. **Please indicate the Country and the CAP/ZIP code where you practice clear aligner treatments:**

    Country: …………………... CAP/ZIP code: …………………

PERCEPTIONS OF CLINICIANS NOT USING CLEAR ALIGNERS

18. **Please, choose one of the following statements better fitting with your perspective:**
    - I used clear aligners in the past but not now
    - I do not use clear aligners and I have no intention to use them
    - I do not use clear aligners but I have the intention to use them in the future
    - I do not know anything about clear aligners

19. **What is your main reason for not using clear aligners?**
    - Clear aligners limit orthodontic treatment outcomes
    - The costs of clear aligners are too high for the provider
    - I have no experience with clear aligners so I prefer not to use them
    - There is no demand for clear aligners in my practice
    - None of the above, please specify: ………………..…

20. **How do you manage your patients requesting clear aligner treatment?**
    - None of the above, please specify
    - You refer them to an orthodontist who uses clear aligners

- You refer them to a general dentist who uses clear aligners
- You refer them to the university or the hospital
- None of the above, please specify: ……………………….................................

DEMOGRAPHIC CHARACTERISTICS, TYPE OF PRACTICE AND BACKGROUND OF THE SURVEY PARTICIPANTS

21. **What is your gender?**
    - Male
    - Female

22. **What is your age?**
    - 24-30 years
    - 31-40 years
    - 41-50 years
    - 51-60 years
    - ≥ 61 years

23. **How many years have you been in practice?**
    - 1-10 years
    - 11-20 years
    - 21-30 years
    - 31-40 years
    - > 40 years

24. **Please indicate the Country and the CAP/ZIP code where you practice clear aligner treatments:**

    Country: …………………...   CAP/ZIP code: …………………
